# Learning and coping strategies versus standard education in cardiac rehabilitation: a cost-utility analysis alongside a randomised controlled trial

**DOI:** 10.1186/s12913-015-1072-0

**Published:** 2015-09-28

**Authors:** Nasrin Tayyari Dehbarez, Vibeke Lynggaard, Ole May, Rikke Søgaard

**Affiliations:** Health Economics, Public Health and Quality Improvement, Olof Palmes Alle 15, 8200 Aarhus N, Denmark; Cardiovascular Research Unit, Regional Hospital West Jutland, 7400 Herning, Denmark; Department of Public Health, Aarhus University, Bartholins Allé 2, Buldg. 1260, 8000 Aarhus C, Denmark; Department of Clinical Medicine, Aarhus University, Bartholins Allé 2, Buldg. 1260, 8000 Aarhus C, Denmark

**Keywords:** Cost-utility analysis, Rehabilitation, Patient education, Ischemic heart disease, Heart failure

## Abstract

**Background:**

Learning and coping education strategies (LC) was implemented to enhance patient attendance in the cardiac rehabilitation programme. This study assessed the cost-utility of LC compared to standard education (standard) as part of a rehabilitation programme for patients with ischemic heart disease and heart failure.

**Methods:**

The study was conducted alongside a randomised controlled trial with 825 patients who were allocated to LC or standard rehabilitation and followed for 5 months. The LC approach was identical to the standard approach in terms of physical training and education, but with the addition of individual interviews and weekly team evaluations by professionals. A societal cost perspective including the cost of intervention, health care, informal time and productivity loss was applied. Cost was based on a micro-costing approach for the intervention and national administrative registries for other cost categories. Quality adjusted life years (QALY) were based on SF-6D measurements at baseline, after intervention and follow-up using British preference weights. Multiple imputation was used to handle non-response on the SF-6D. Conventional cost effectiveness methodology was employed to estimate the net benefit of the LC and to illustrate cost effectiveness acceptability curves. The statistical analysis was based on means and bootstrapped standard errors.

**Results:**

An additional cost of DKK 6,043 (95 % CI −5,697; 17,783) and a QALY gain of 0.005 (95 % CI −0.001; 0.012) was estimated for LC. However, better utility scores in both arms were due to higher utility while receiving the intervention than better health after the intervention. The probability that LC would be cost-effective did not exceed 29 % for any threshold values of willingness to pay per QALY. The alternative scenario analysis was restricted to a health care perspective and showed that the probability of cost-effectiveness increased to 62 % over the threshold values.

**Discussion:**

The LC was unlikely to be cost-effective within 5 months of follow-up from a societal perspective, but longer-term follow-up should be evaluated before a definite conclusion is drawn.

**Conclusion:**

Future research should assess the LC strategies' long-term efficacy and cost-utility.

**Trial registration:**

NCT01668394

## Background

Cardiac rehabilitation (CR) programmes have become an integral part of the standard of care in modern cardiology. These programmes rely on early detection of the disease process and the application of interventions to prevent disease progression [1]. However, few patients complete CR or succeed with lasting lifestyle improvements [2]. Despite the established value of CR, the participation rates are disappointingly low [3], and there is a lack of visibility and recognition of the importance of CR by the public [4].

In the light of the favourable effects of CR, it is important to develop patient education strategies which can help patients to improve adherence to CR and make changes towards a healthier lifestyle [5, 6]. To enhance patient attendance in the cardiac rehabilitation programme, a patient-education strategy called “Learning and Coping (LC)” was implemented within a randomised controlled trial (RCT) at the Regional Hospitals West Jutland in Denmark. This method was developed in Norway to address some of the challenges in patient education. Theoretical basis and study protocol of LC strategies has been published before [7, 8].

Economic evaluation of CR has been reported since the 1980s, and all of the studies have supported the implementation of CR [9]. The cost-effectiveness of CR is supported by evidence from 15 health economic evaluations conducted in North America and Europe [10]. Recent studies have focused on comparison among various modes of delivery of CR, such as programmes that were outpatient, inpatient, home-based or telephone coaching programmes [9, 11].

The aim of this study was to evaluate whether a LC method was cost-effective compared with the standard rehabilitation of patients with ischemic heart disease (IHD) and heart failure (HF).

## Methods

### Study design and population

The study was conducted alongside a RCT of patient education in cardiac rehabilitation. The participants in this trial included 827 patients over 18 years who had been admitted with IHD or HF. Two patients were excluded; one because he was mistakenly randomised twice and the other because she was due for an eye operation and could not start the CR program. A total of 825 patients undergoing the CR programme, were randomised in to one of two education methods. Randomisation was computer generated and stratified by hospitals, diagnosis and gender. Use of stratified randomisation should be viewed as an insurance policy against a potential imbalance and, because it has virtually no cost, it should be routinely used in RCTs [12]. Of these patients, 413 were randomised to the LC group and 412 to the control group. All of the patients received 8 weeks (approximately 2 months) of training and education and were followed for an additional 3 months.

### Intervention

In this study, LC strategies were applied and compared with the standard education in CR at three hospitals in the western part of Central Denmark Region (Table 1). Detailed components of education strategies is available in the study protocol [8].

**Table 1:** Elements of educational strategies in cardiac rehabilitation

Learning and coping	Standard
- Initial individual clarifying interview	
- 8 week group-based:	- 8 week group-based:
Physical training 1½ h × 3/week	Physical training 1½ h × 3/week
Education 1½ h/week	Education 1½ h/week
- Weakly team evaluation	
- Final individual clarifying interview	

### Costs

We assessed costs from the societal perspective to estimate the long-term average costs of the routine provision of education. A micro-costing approach was used to calculate the cost of intervention. Micro costing is a method that provides crucial and detailed cost data. An accurate cost of the intervention at the micro-level is required to perform accurate economic analysis, such as cost-effectiveness or cost-benefit analyses, which gives a complete analysis of outcomes alongside the cost at which they are achievable [13].

The cost of implementing the intervention was not included in the present analysis; it should be seen as estimating the long-term average costs of implementing the intervention in routine practice. The intervention includes the cost of both formal and informal time of all persons involved.

A load factor of 1.5 was applied to the cost of health professional’s formal time to account for non-productive time. Their productive time was assumed to amount to 45 min of an hour (load of 0.25) due to pauses, walking distance between locations, private time, etc. The remainder load (0.25) was considered to include vacation, sickness, participation in seminars and educational courses etc.

The valuation of formal care was based on the average gross salary of nurses and physiotherapists involved in patient education, which was provided by Regional Hospital West Jutland. Regional hospital’s standard overhead rate of 21 % was applied to account for capital costs.

The valuation of informal time; time spent by patients and expert patients, was undertaken using the opportunity cost method, in which the value of a person’s time is reflected by his or her wage rate. The average age of expert patients was 65 years, and the valuation of their time was based on their net salary assuming they are pensioner. National average gender- and age-matched salaries were used to value the leisure time (net salary) and productive time (gross salary) [14].

The patient’s time was calculated based on the recorded data on the number of training and education sessions they attended in both arms.

Transportation cost was assumed to be 20 min and 10 km each way for all patients and all contacts. The government tariff for transportation by private car for 2013 was used. Regional Hospital West Jutland paid the transportation cost of the expert patients during the intervention. To value the transportation time of the expert patient, 20 min in each way was assumed (Table 2).

**Table 2:** Item costs used for estimating the costs of patient education in cardiac rehabilitation programme

Resources	Cost	Source
Time cost of formal care		
Nurse (DKK /hour)	197	Regional Hospital West Jutland
Physiotherapist (DKK/hour)	160	Regional Hospital West Jutland
Load-factor for personnel time (weight)^a^	1.5	Researcher assumption
Expert patient, 65 years old, net salary (DKK/hour)	108	Statistics Denmark
Capital cost	21 %	Hospital standard rate
Time cost of informal time (examples)^b^		
Male, 60-64years old, gross salary (DKK/hour)	164	Statistics Denmark
Male, 60-64years old, net salary (DKK/hour)	144	Statistics Denmark
Female, 60-64years old, gross salary (DKK/hour)	134	Statistics Denmark
Female, 60-64years old, net salary (DKK/hour)	111	Statistics Denmark
Transportation cost, expert patients (DKK/way)	49	Regional Hospital West Jutland
Transportation cost, patients (DKK/kilometres)	3.82	Danish Government

^a^A load-factor was applied to account for non-productive time of therapist and other absence from work

^b^Complete set of age- and gender- specific national average salaries was used (available at http://statistikbanken.dk)

The DREAM database, which contains information on all social benefits, was searched for events of inability to work. Productivity losses are due to sickness leave, early pension and re-schooling and, were calculated using weeks of inability to work for those who did not reach the age for pension during follow-up (67 years old). All cost estimates were adjusted for time preference and, inflated to the common price year of 2013, using the consumer price index where relevant (DKK 100 ≈ GBP 10 ≈ USD 18).

Data on primary health care use (number of visits and the related activity-based tariffs) were extracted from the National Health insurance service register [15], and the data on the use of secondary health care services (number of services and national average Diagnostic-related grouping (DRG) tariffs) were extracted from the National Patient Registry (NPR) [16]. The data for prescribed medicine were extracted from the national prescription registry [17]. The DRG is revised every year and is based on the average use of resources in each group. The NPR includes administrative information, diagnoses, diagnostic and treatment procedures using several international classification systems, including the International Classification of Disease and Related Health Problems 10^th^ revision [18].

### Outcome parameter

The outcome parameter in the study was health-related quality of life, as defined by the SF-6D. The SF-6D scores were preference-weighted using British weights [19]. QALY was estimated as the area under the health utility curve over time using linear interpolation between observations [20].

### Imputation

The data suffered missing values on the SF-6D scores, while complete data on cost were obtained from administrative national registers with full coverage and thus were not subjected to imputation. To avoid the loss of information on the outcome parameters, the missing values were replaced using multiple imputation, which is generally used to address data missing at random [21].

Imputation was produced using a chained equation approach [22] because the non-response was of a non-monotonic character (e.g., a non-response at baseline could return and become a response after intervention or follow-up). Furthermore, sensitivity analysis was conducted for the alternative analytical choice of carrying the baseline observation to impute missing values after the intervention and carry the after intervention observation to impute missing values on the 3-month follow-ups.

The data on patient attendance at education sessions were not registered in the first six months of the study. In this case, missing data was completely random and did not depend on observed or unobserved values [23], and to avoid loss of information, the missing values were imputed by a mean within the randomisation groups.

### Statistical methods

Baseline characteristics were summarised using conventional summary statistics. The comparative analysis of the individual parameters and of the net benefit was based on the means with bootstrapped standard error [24]. Non-parametric bootstrapping with 10,000 replications was applied due to the skewed nature of both resource use and cost, and a general significance level of 0.05 was used.

The analytical strategy was implemented for two scenarios: cases with a complete response of the outcome parameter and, an imputed dataset in which missing values of the outcome parameter were imputed. Although both scenarios are shown, the latter was considered the main analysis.

Economic evaluation is subject to uncertainty not only because of sample variation but also because of assumptions made and generalisability issues [25]. We therefore conducted an analysis of alternative scenarios to test the methodological uncertainty of the imputation procedure and costs. The impact of the alternative scenarios was also illustrated using cost-effectiveness acceptability curves. All analyses were conducted in STATA version 13.

### Cost-utility evaluation

We estimated the net benefit using a range of hypothetical threshold values for decision-makers’ willingness to pay for a QALY (from DKK 0 to DKK 500000) and presented the incremental cost and incremental effects visually in a so-called cost-effectiveness acceptability curve (CEAC). The CEAC was used to illustrate the probability of the intervention being cost-effective for the range of threshold values for willingness to pay for a QALY [26, 27]. These curves illustrate the probability that an intervention (LC) is cost effective compared with the control (standard) for a continuum of hypothetical threshold values of willingness to pay for a QALY.

### Ethics

Approval from Central Denmark Region ethics committee is obtained (journal number 20100230) and all participants provided written informed consent.

## Results

A total of 825 patients were included in the study. A summary of the patients’ baseline characteristics is presented in table 3, and no significant baseline differences in age, gender and diagnosis between the groups were found. Patients in the LC group participated in significantly more training and education sessions than did participants in the control group.

**Table 3:** Population characteristics

	Learning and coping (*n* = 413)	Standard (*n* = 412)
Age at randomisation, mean (range)	63 (33–92)	63 (27–89)
Male gender, n (%)	313 (76)	312 (76)
Diagnosis		
Ischemic heart disease, n (%)	326 (79)	323 (78)
Heart failure, n (%)	87 (21)	89 (22)
Adherence to programme sessions		
Physical training, mean (range)*	19.61 (1–24)	18.48 (1–24)
Education, mean (range)*	6.46 (0–9)	5.97 (0–9)

The asterisks indicate statistically significant differences

### Intervention cost

Table 4 details the costs of the intervention using a micro-costing approach based on an average number of 10 patients in each course. Because there was no registration of the exact duration of each session, the planned 1.5 h of education and training and 1 h for the interview and weekly team evaluation were applied. The provision of LC in CR was estimated to incur an additional intervention cost of DKK 2072 compared with the standard method for one patient.

**Table 4:** Cost of cardiac rehabilitation programme (DKK)

	Learning and coping		Standard	
	Number of units	Total cost	Number of units	Total cost
Nurse				
Training sessions (hours)	36	7092	36	7092
Education sessions (hours)	8	1576	8	1576
Interviews (hours)	10	1970	0	0
Weekly team evaluation (hours)	8	1576	0	0
Load for unproductive time	31	6107	22	4334
Total	93	18,321	66	13,002
Physiotherapist				
Training sessions (hours)	36	5760	36	5760
Education sessions (hours)	4	640	4	640
Interviews (hours)	10	1600	0	0
Weekly team evaluation (hours)	8	1280	0	0
Load for unproductive time	29	4640	20	3200
Total	87	13,920	60	9600
Expert patient				
Training sessions (hours)	12	1296	0	0
Education sessions (hours)	12	1296	0	0
Weekly team evaluation (hours)	8	864	0	0
Transportation time (hours)	16	1728	0	0
Transportation^a^	Not applicable	2304	0	0
Total	48	7488	0	0
Overhead		8343		4746
Total cost for 10 patients		48,072		27,348
Average cost per patient		4807		2735

^a^Transportation cost of expert patients was paid by hospitals. The average for one course was calculated

### Resource utilisation and cost

Tables 5 and 6, show the mean resource utilisation and cost, respectively. Patients in the LC arm used more health resources in primary healthcare, medicine prescription and outpatient visits, but only the difference in outpatient visits was statistically significant (*p* = 0.002).

**Table 5:** Resource use during 5 months of follow-up^a^

	Learning andcoping	Standard	Difference
	(*n* = 413)	(*n* = 412)	(CI 95 %)
Primary health care (contacts)			
General practice	13.58 (0.56)	12.93 (0.58)	0.65 (−0.94;2.23)
Medical specialist	0.93 (0.10)	0.90 (0.11)	0.03 (−0.26;0.32)
Physiotherapist	1.98 (0.39)	1.84 (0.35)	0.14 (−0.89;1.16)
Dentist	1.66 (0.10)	1.49 (0.09)	0.17 (−0.09;0.44)
Other	0.09 (0.03)	0.09 (0.03)	0.005 (−0.07;0.08)
Prescribed medicine	15.46 (0.59)	14.58 (0.68)	0.89 (−0.87;2.65)
Secondary health care			
Outpatient visits*	26.61 (0.43)	24.51(0.50)	2.10 (0.80;3.41)
Hospital bed days	1.54 (0.34)	1.38 (0.29)	0.17 (−0.71;1.05)
Hospital admissions^b^	0.49 (0.06)	0.57 (0.08)	−0.09 (−0.28;0.11)
Sick-leave weeks	4.69 (0.44)	4.54 (0.44)	0.15 (−1.07;1.37)
Re-schooling^c^	0.02 (0.02)	0.05 (0.05)	−3.03 (−0.15;0.08)
Disability pension	2.12 (0.31)	1.60 (0.28)	0.52 (−0.30;1.33)
Sick Weeks	2.13 (0.31)	2.33 (0.31)	−0.2 (−1.06;0.66)
Informal time of patients			
Patient time in course (hours)*	40.92 (0.64)	36.68 (0.76)	4.23 (2.28;6.18)
Patient time in transportation (hours)	18.59 (0.28)	16.30 (0.34)	2.28 (1.42;3.14)
Patient transportation (kilometre)	557.71 (8,68)	489.13 (10.28)	68.58 (42.39;94.77)

^a^Values are mean (SE), unless otherwise stated

^b^Hospital admissions account for average number of admissions to the different hospital wards

^c^Re-schooling refer to a situation in which patient is learning new skills because he/she is not able to stay in the current job

The asterisks indicate statistically significant differences

**Table 6:** Mean cost (SE) during 5 months of follow-up (DKK)

	Learning and coping	Standard	Difference
	(*n* = 413)	(*n* = 412)	(CI 95 %)
Intervention^a^	4807	2735	2072
Primary health care			
General practice	1361 (6042)	1314 (66.33)	47 (−128.50;221.89)
Medical specialist	252 (33.16)	234 (35.65)	17 (−77.51;112.57)
Physiotherapist	120 (28.35)	112 (26.01)	8 (−66.59;82.49)
Dentist	186 (14.38)	160 (10.30)	26 (−8.67;60.52)
Other	28 (9.47)	31 (12.60)	−4 (−34.93;26.10)
Total	1947 (81.49)	1853 (86.85)	94 (−140.80;329.06)
Prescribed medicine	2064 (95.18)	2022 (108.45)	42 (−239.60;323.62)
Secondary health care			
Outpatient visits	44,050 (800.01)	41,513 (1018.42)	2537 (−16.31;5090.90)
Hospital admissions	17,644 (3056.81)	19,250 (3027.52)	−1608 (−10091.22;6875.10)
Total	61,692 (3171.31)	60,763 (3172.60)	929 (−7863.18;9721.64)
Productivity loss			
Re-schooling	116 (114.41)	242 (237.63)	−126 (−679.55;428.17)
Disability pension	13,392 (2030.54)	10,336 (1847.17)	3056 (−2290.51;8402.31)
Sick Weeks	14,450 (2120.91)	15,660 (2144.589)	−1210 (−7203.09;4783.19)
Total	27,958 (2738.37)	26,238 (2708.10)	1720 (−5885.65;9326.14)
Informal time			
Patient time in course	5354 (95.36)	4752 (105.19)	602 (321.84;881.86)
Patient time in transportation	2433 (42.35)	2112 (47.08)	321 (196.77;444.68)
Total	7788 (134.64)	6864 (153.19)	923 (523.95;1322.82)
Patient transportation	2130 (32.34)	1868 (38.88)	262 (162.93;361.03)
Total costs	108,388 (4245.75)	102,345 (4199.11)	6043 (−5697.02;17783.36)

^a^Measuring confidence interval for intervention is not applicable

The number of sick-leave weeks due to disability pension was higher in the LC arm, and it was higher in the standard arm due to re-schooling and sick leave. However, these differences were not statistically significant.

Patient time was statistically significantly higher in the LC arm due to participation in clarifying interviews at the beginning and the end of the intervention in addition to more adherence to training and education sessions.

The total societal costs of the intervention and caring for a patient with IHD or HF were estimated to average DKK 108,388 and DKK 102,345 in the LC and standard arms, respectively.

### Health outcomes

The analysis that was based on the completed response on out-come parameter at all steps consisted of 58.19 % cases in the LC arm and 54.42 % in the control arm.

Due to reduction in the number of respondents after the intervention and follow-up, multiple imputation was used. A non-response analysis was conducted for age, gender, primary health care cost, secondary health care cost, medicine cost, production loss cost and the number of attended education and training sessions for missing values in the effect parameters. Statistical significance was observed for all variables except age, gender, primary health care cost and intervention cost. The identified predictors of non-responses were used in the imputation procedure on which the main analysis was based. The extent and impact of imputation is shown in table 7. The imputed values for the SF-6D scores were generally lower than the observed values, which indicates that responders were generally better off than non-responders. However, better utility scores in both arms were due to higher utility while receiving the intervention than better health after the intervention. No statistically significant differences in SF-6D scores were observed between the groups.

**Table 7:** QALY during 5 months of follow-up

	Learning and coping			Standard			Difference
	n	Mean	(SE)	n	Mean	(SE)	(CI 95 %)
Complete response-based analysis							
SF-6D							
Baseline	238	0.739	0.008	223	0.723	0.008	0.016 (−0.006;0.039)
After intervention (2 months)	238	0.798	0.007	223	0.780	0.008	0.018 (−0.003;0.040)
After follow-up (5 months)	238	0.794	0.008	223	0.792	0.008	0.002 (−0.020;0.025)
QALY	238	0.327	0.003	223	0.322	0.003	0.005 (−0.003;0.014)
Multiple imputation-based analysis							
SF-6D							
Baseline	413	0.720	0.006	412	0.705	0.006	0.014 (−0.003;0.032)
After intervention (2 months)	413	0.771	0.006	412	0.754	0.006	0.016 (−0.001;0.034)
After follow-up (5 months)	413	0.788	0.006	412	0.782	0.006	0.006 (−0.011;0.023)
QALY	413	0.319	0.002	412	0.314	0.002	0.005 (−0.001;0.012)

### Cost-utility

**Fig. 1:**
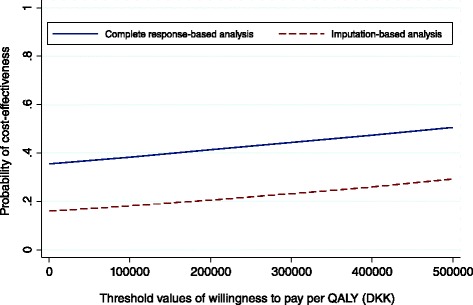
Cost-effectiveness acceptability curve: main analysis

Because the intervention did not seem to be cost saving, the potential for cost-effectiveness was limited. Figure 1 shows the probability of the intervention being cost-effective on a continuum of hypothetical threshold values for decision-makers willingness to pay for an additional QALY. The curve improves slightly for increasing threshold values of willingness to pay due to a slight increase in QALY gained by LC. The probability for cost-effectiveness did not exceed 29 % for the imputation-based analysis and 51 % for complete-response based analysis over the range of threshold values.

**Fig. 2:**
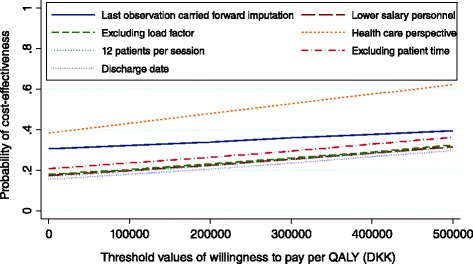
Cost-effectiveness acceptability curve: sensitivity analysis

Figure 2 shows how cost-effectiveness changes for several scenarios regarding imputation and various cost alternatives. Examining the cost from the health care provider’s perspective increased the probability of cost-effectiveness to 62 % over threshold values. Cost-effectiveness was not markedly different across various cost scenarios, including therapists with lower salaries (DKK 150 per hour for a nurse and DKK 120 per hour for a physiotherapist), excluding the load factor and using a maximum number of 12 patients per session. Excluding patients time resulted in the probability of cost-effectiveness reaching 36 % over the threshold values.

Because the discharge date from the hospital for some patients occurred after the follow-up period, we performed a sensitivity analysis that excluded these admissions from the study. This sensitivity analysis resulted in the probability of cost-effectiveness reaching 30 % over the threshold values.

## Discussion

The present economic evaluation examined the cost-utility of patient education in CR and was designed to assist health care providers in determining which education strategy should be provided. The main finding was that LC is unlikely to be cost-effective because it led to a higher average usage of all care categories except inpatient care, and the additional QALY generated was not statistically significant. The LC education was also associated with higher costs compared with standard education. This higher cost was primarily driven by higher outpatient costs, productivity loss costs and patient time costs.

The lower rate of hospital admissions in the LC arm could be due to more effective education, which may have led to seeking medical interventions earlier in the disease process. Because we analysed health care utilisation during a limited period of 5 months, the longer-term health care utilisation and costs are unclear.

To the best of our knowledge, this is the first report on cost-utility of various patient education strategies in CR of patients with IHD or HF in Denmark. In a review of patient education in the management of coronary heart disease, James P.R. Brown stated that five studies reported healthcare utilisation and costs, but no study reported on cost-effectiveness. Reflecting on the various education modalities and intensities of the intervention, the reported cost of provision per patient varied from 49 to 453 lb. He found no strong evidence that education reduced all-cause mortality, cardiac morbidity, revascularisation or hospitalisation compared with the control. However, there is some evidence that patient education may be cost saving compared with usual care because of a reduction in downstream healthcare utilisation [28].

In a systematic review on patient education strategies for hospitalised cardiovascular patients, Yvonne Commodore-Mensah stated that it is unclear which educational intervention elements or strategies are most effective for educating hospitalised cardiovascular patients and their families. Her review showed that there are various patient education strategies that can be implemented for hospitalised cardiovascular patients and families; however, interventions need to be feasible as well as cost-effective [29].

In a recent study in Australia, Sangster et al. compared the cost-effectiveness of a telephone-delivered Healthy Weight intervention to a telephone-delivered Physical Activity intervention and reported an average gain of 0.007 additional QALY and a difference of $852 in cost, both in favor of the Healthy Weight intervention.

We did not find a significant improvement in health status as assessed with the SF-6D in both the LC and standard groups. Additional studies have also indicated that if the follow-up period is 6 months or less, no significant improvement in quality of life or functional status of patients with coronary heart disease is found [30]. A systematic review and meta-analysis performed by Rod S. Taylor on exercise-based rehabilitation for patients with coronary heart disease identified 12 trials that assessed health-related quality of life using a range of outcome measures. Although all trials demonstrated an improvement in quality of life with cardiac rehabilitation, an improvement was also reported consistently in control patients [31].

### Strengths and weaknesses of the study

Complete data on the costs derived from the national registries was one of the strong aspects of this study [15–17]. Additionally, using the SF-6D is suitable to measure health-related quality of life [32]. Another strength of the study was that rather than ignoring non-responders, we chose to use a multiple imputation strategy. The validity of the imputation procedure was based on the assumption that the data were missing at random.

## Conclusion

In this study, we demonstrated that there were no significant differences in either costs or outcomes between LC and standard education methods from a societal perspective during 5 months follow-up. We concluded that LC was not a cost-effective intervention in the short term; however, analysing a longer period of follow-up seems necessary because a higher cost of outpatient care in LC may result in future cost saving. Therefore, it could be suggested that health problems may be identified at an earlier stage, thereby resulting in better health outcomes.
